# Multi-scale semantic enhancement network for object detection

**DOI:** 10.1038/s41598-023-34277-7

**Published:** 2023-05-03

**Authors:** Dongen Guo, Zechen Wu, Jiangfan Feng, Tao Zou

**Affiliations:** 1 School of Computer and Software, Nanyang Institute of Technology, 80 Changjiang Road, Nanyang, 473004 Henan China; 2 Chongqing Engineering Research Center for Spatial Big Data Intelligent Technology, Chongqing University of Posts and Telecommunications, No. 2, Chongwen Road, Chongqing, 400065 Chongqing China

**Keywords:** Computer science, Information technology

## Abstract

In the field of object detection, feature pyramid network (FPN) can effectively extract multi-scale information. However, the majority of FPN-based methods suffer from a semantic gap between features of various sizes before feature fusion, which can lead to feature maps with significant aliasing. In this paper, we present a novel multi-scale semantic enhancement feature pyramid network (MSE-FPN) which consists of three effective modules: semantic enhancement module, semantic injection module, and gated channel guidance module to alleviate these problems. Specifically, inspired by the strong ability of the self-attention mechanism to model context, we propose a semantic enhancement module to model global context to obtain the global semantic information before feature fusion. Then we propose the semantic injection module to divide and merge global semantic information into feature maps at various scales to narrow the semantic gap between features at different scales and efficiently utilize the semantic information of high-level features. Finally, to mitigate feature aliasing caused by feature fusion, the gated channel guidance module selectively outputs crucial features via a gating unit. By replacing FPN with MSE-FPN in Faster R-CNN, our models achieve 39.4 and 41.2 Average precision (AP) using ResNet50 and ResNet101 as the backbone network respectively. When using ResNet-101-64x4d as the backbone, MSE-FPN achieved up to 43.4 AP. Our results demonstrate that replacing FPN with MSE-FPN significantly enhances the detection performance of state-of-the-art FPN-based detectors.

## Introduction

Deep learning has demonstrated significant progress in image classification and object detection owing to its powerful feature learning and transfer learning capabilities. A series of detectors (Fast R-CNN^1^, FasterR-CNN^2^, YOLO^3^, SSD^4^, Cascaded R-CNN^5^, RetinaNet^6^) have been proposed to obtain significant performance improvements. Among these, FPN^7^ is a framework for object detection that can significantly increase the performance of object detection models. Specifically, the final feature maps at various scales contain rich semantic information because low-level feature maps in FPN combine semantic information from high-level feature maps of various scales. FPN-based methods have produced an outstanding performance in object detection by combining feature from various scales. However, FPN has three pervasive limitations: (1) features channel information lost during dimensionality reduction, (2) a significant semantic gap between features at various levels, (3) the feature confusion caused by multi-scale fusion. Although existing methods such as Libra R-CNN^8^, AugFPN^9^, and CEFPN^10^ can alleviate these issues to some extent, there is still ample room for improvement, we will describe these issues in the following.

**Figure 1:**
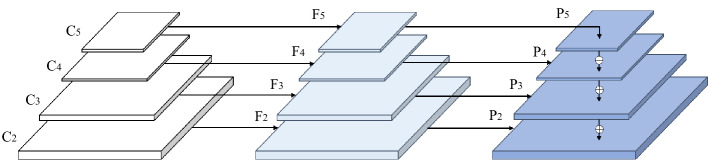
Feature pyramid network (FPN) structure. Where $C_i$ represent the original feature layers, $F_i$ represent the feature layers after dimensionality reduction using 1 $\times$ 1 convolution, and $P_i$ represents the feature layer used for prediction after feature fusion.

### Advanced feature map channel information lost

As shown in Fig. 1, FPN-based methods typically employ a $1\times 1$ convolutional layer between $C_i$ and $F_i$ to perform dimensionality reduction. However, when dimensionality reduction is performed on advanced features with rich semantic information (the number of channels changes from 2048 to 256), the channel information may be lost, and this lost information could be as critical to the detection task as the retained information. To address this issue, the existing method BiFPN^11^ performs multiple weighted feature fusions between $F_i$ and $P_i$ to enhance the feature expression ability of the prediction layer, which achieves higher detection accuracy but brings a lot of computation; AugFPN^9^ adds a residual feature enhancement module between $C_i$ and $P_i$ to reduce the information loss after channel reduction. However, these methods focus on reducing channel loss due to dimensionality reduction before feature fusion and do not take full advantage of the rich semantic information in $C_i$.

### Semantic gaps between features at different levels

In FPN, low-level features are transferred layer by layer through the backbone network to obtain high-level features. Low-level features contain rich spatial information, which is useful for detecting the position of objects in the image, but have poor semantic information, making it challenging to determine the class of detected objects. High-level features, on the other hand, have rich semantic information but less spatial information, making it difficult to pinpoint the precise location of objects. Therefore, low-level and high-level features complement each other in object detection. To reduce the number of feature channels, features at various levels were processed through a simple $1\times 1$ convolutional layers prior to feature fusion. Directly fusing these features while ignoring the significant semantic gaps between features weakens the expressiveness of multi-scale features, resulting in dilution of semantic information during top-down feature fusion. To solve this problem, PAFPN^12^ adopts top-down and bottom-up fusion methods to effectively exchange information between high-level features and low-level features. Libra R-CNN^8^ solves the problem of unbalanced semantic information during training by fusing high-level features and low-level features through a balanced feature pyramid.

### Multi-scale fusion leads to feature confusion

Cross-scale fusion (Libra R-CNN^8^) and skip linking (BiFPN^11^) techniques have been successful in improving the performance of FPN-based object detection models. However, there is a semantic gap between feature maps at different scales, and immediate fusing following linear interpolation may result in feature confusion (FPN^7^), stacking multiple integrated features may loss location information, resulting in localization and recognition task confusion (ACFPN^13^).

To overcome these challenges, we present three novel components in this paper. First, inspired by the self-attention mechanism, we introduce a semantic enhancement module with channel-spatial attention, which resizes feature maps of different scales to a uniform size and concatenates them along the channel dimension to obtain the global context, the global context is then modelled using attention mechanisms to obtain global semantic information. Second, we propose semantic injection modules for reducing the semantic gap between features at different scales. Third, inspired by the gating unit^14^, we introduce a gated channel guidance module to reduce feature confusion caused by feature fusion. We name our whole model as Multiscale Semantics Enhance Feature Pyramid Network (MSE-FPN), which is flexible and generalisable for FPN-based detectors. Our main contributions are as follows:

1. Inspired by the self-attention mechanism, we propose a semantic enhancement module with channel-spatial attention to generate global semantic information and introduce a semantic injection module to reduce the semantic gap between feature maps at different scales.
2. To reduce the confusion of features at different scales, a simple and effective gated channel guidance module is introduced before feature fusion.
3. We evaluated our proposed object detection framework on MS COCO and showed that it outperformed the FPN-based detector significantly.

## Related works

### Model-based methods to object detection

With the advent of deep convolutional networks, object detection has advanced significantly in recent years. Current object detection methods follow a one-stage and two-stage model. R-CNN^15^ was the first to use selective search to generate region suggestions, combining a convolutional neural network with object detection. SPPNet^16^ and Fast R-CNN^1^ use R-CNN to extract feature maps for the entire image and then use spatial pyramidal pools and ROI pools to generate region features, respectively. The region proposal network (RPN) was proposed by Faster R-CNN^2^ to improve detector performance and enable end-to-end training of the detector. Since then, many methods have improved R-CNN^15^ from different angles. For example, to handle the problem of multi-scale detection, FPN^7^ realizes prediction from different levels through the pyramid structure and solves the scale change. Cascade R-CNN^5^ is a classic yet powerful cascaded architecture that extends Faster R-CNN^2^ to multi-stage detectors. Mask R-CNN^17^ extends FasterR-CNN^2^ by adding a mask branch to flexibly adapt to multiple detection tasks. In one-stage detectors, localization and classification are usually achieved directly using a unified network, which achieves higher efficiency but loses some accuracy to a certain extent. SSD^4^ can detect objects of different scales. YOLO^3^ uses feature maps to predict object categories and regression boxes. RetinaNet^6^ relies on focal loss to overcome the problem of significant imbalance in the ratio of positive to negative samples in one-stage object detection to improve accuracy.

### Semantic gaps in multi-scale features

Different scale features have significant semantic differences, and direct fusion may result in feature misunderstanding. There is a top-down path put forward by Feature Pyramid Network (FPN) to combine multi-scale features. Following this idea, PAFPN^12^ adds an additional bottom-up path aggregation network on top of FPN; Libra R-CNN^8^ proposes a balanced pyramid, which fuses features from all levels and uses a self-attention mechanism^18^ to refine balanced semantic features; EfficientDet^11^ presents a weighted bidirectional FPN To conduct feature fusion; AugFPN^9^ implements consistent supervision to close semantic gaps between features at various size; CEFPN^10^ uses sub-pixel convolutional for downsampling to reduce the semantic gap between different scales. AC-FPN^13^ adaptively captures semantic and localization information using an attention-guided module to enhance the discriminative ability of feature representations. MIDF^19^ uses a novel remote sensing text-image retrieval (RSCTIR) framework based on global and local information, and design a multi-level information dynamic fusion (MIDF) module to efficaciously integrate features of different levels. MIDF leverages local information to correct global information, utilizes global information to supplement local information, and uses the dynamic addition of the two to generate prominent visual representation. MCRN^20^ uses a multi-source cross-modal retrieval network (MCRN) based on contrast learning and generative adversarial networks, the designed model establishes a shared feature space through modal entanglement and multimodal shared encoding, which in turn yields a common representation of multiple information sources at the semantic level. In contrast to the above, we propose semantic enhancement module and semantic injection module cooperate to solve this problem. Inspired by the attention-guided module^13^, we propose a channel-spatial attention mechanism. Unlike the attention-guided module, channel-spatial attention has two branches, one of spatial-attention uses the powerful modeling capability of self-attention to model the global context to obtain global semantic information, and the other channel-attention branch applies weighting to the global channel information in the context, which effectively reduces the channel information lost due to $1 \times 1$ convolutional downscaling. We apply channel-spatial attention to the original feature layer and name it semantic enhancement module(SEM). SEM focuses on augmenting the original features to retain as much semantic and channel information as possible that is beneficial to the detection task. To prevent the rich semantic information being thinned in the top-down fusion structure, we directly up-sample the global semantic information and fuse it with the original feature map, which is called the semantic injection module.

### Feature confusion optimization

Alleviating cluttered feature fusion is key to realizing the full potential of the model structure. Libra R-CNN^8^ performs a refinement operation after feature fusion to reduce feature confusion; CEFPN^10^ weights the fused features and maps the weights to each feature when generating different levels of features to optimize the final feature when generating different levels of features; AugFPN^9^ proposes a method to adaptive fuse all levels of features using a set of learnable parameters; CBAM^21^ applies feature refinement using the channel attention module (CAM) and the spatial attention module (SAM), and achieves significant performance improvements while keeping the overhead small; Inspired by CAM^21^, CEFPN^10^ uses channel attention guided module (CAG) weights the fused features when generating different levels of features and maps the weights to each feature to optimize the final features when generating different levels of features; coordinate attention (CA)^22^ uses a separate module to aggregate information along spatial and channel directions, embedding positional information into channel attention, inheriting the benefits of channel attention while capturing long-range dependencies with precise positional information. GMU^23^ uses a strategy to learn fusion transformations from multimodal sources, in synthetic experiments the GMU was able to learn hidden latent variables, and in a real scenario it outperformed the singlemodality approaches. Unlike the aforementioned methods, inspired by gating units^14^, we introduced gating units into the gated channel guidance module to capture internal dependencies in feature maps and reduce feature confusion. Specifically, we weighted the fusion of features at different levels before feature fusion, using gating units to selectively receive information from features at different scales to mitigate feature confusion.

## Methodology

**Figure 2:**
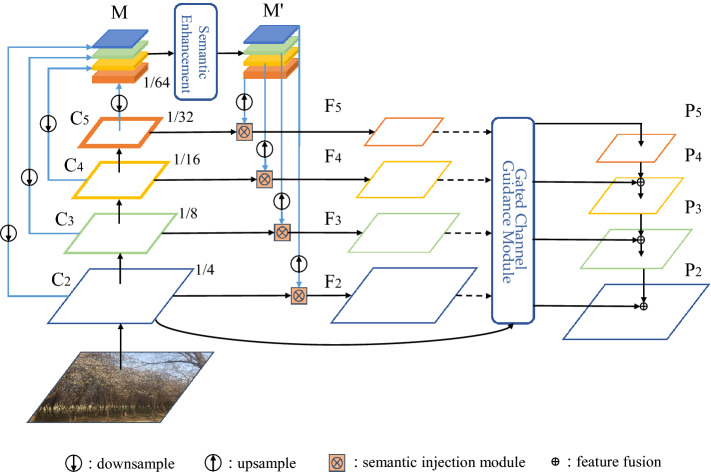
An overview of our MSE-FPN. The semantic enhancement module (SEM) is used to extract and integrate global semantic information. Semantic injection module (SIM) maps global semantic information features to corresponding levels. Gated channel guidance module (GCG) performs feature fusion after weighted optimization of features.

The overall structure of MSE-FPN is shown in Fig. 2. Our goal is to use the semantic enhancement module and semantic injection module to narrow the semantic gap between different feature layers to obtain better feature fusion. Moreover, we employ the gated channel guidance module to alleviate the confusion effect after feature fusion. We describe these three modules in detail below.

### Semantic enhancement module

In FPN^7^, the residual network^24^ is usually utilized as the backbone network to construct feature maps with different resolutions $\{256, 512, 1024, 2048\}$. The lower-resolution images $\{C_4, C_5\}$ contain rich semantic and channel information, while the higher-resolution images $\{C_2, C_3\}$ contain rich spatial information. As shown in Fig. 1, the top-down fusion strategy of FPN gradually thins out the rich semantic information contained in high-level feature layers. Moreover, the simple $1\times 1$ convolution for dimensionality reduction in obtaining $F_i$ from $C_i$ leads to the loss of some channel information, which makes the rich channel information of $C_i$ not fully utilized, and this lost channel information and retained channel information may have the same importance for detection tasks.

To tackle the above problems, we propose a semantic enhancement module (SEM) to obtain global context by concatenating features at different levels, integrating and refining different spatial and channel information to make full use of information from the most original feature layer $C_i$. We use channel-spatial attention to model the spatial information of the global context to extract rich semantic information, weight the channel information of the global context to decrease information loss in the $F_i$ channel. The input to the SEM is the global context *M*, which is obtained by downsampling the original feature layers ${C_2, C_3, C_4, C_5}$ in the backbone network to a uniform scale and concatenating them along the channel dimension. We use an adaptive averaging pool to decrease all feature maps at different levels to 1/64 of the input size when downsampling, effectively reducing the computational effort without much loss of accuracy. The shape of the global context *M* is $C_n \times H \times W$, N denotes the number of layers in the original feature layers $\{C_2, C_3, C_4, C_5\}$, thus N=5, the size of $C_n$ is:

1 $$\begin{aligned} C_n= & {} \sum _{i=2}^N C_i, \end{aligned}$$

2 $$\begin{aligned} M= & {} concat(downsample(C_2,C_3,C_4,C_5)), \end{aligned}$$

Specifically, channel-spatial attention has two branches. The first branch is the channel-attention branch that weights the channel information in the global context, a global average pooling of the global context *M* is used to generate a $1 \times 1 \times C$ feature map, then use two convolutions to exchange channel information and sigmoid it to obtain the channel weight $M_w$, this process can be expressed as

3 $$\begin{aligned} M_w=\sigma (FC_1(\delta (FC_2(AvgPool(M))))), \end{aligned}$$

which $\sigma$ represents the sigmoid function and $\delta$ refers to the ReLU function, $FC_1$ and $FC_2$ represent a fully connected operation.

The second branch is spatial-attention branch, which uses the powerful modelling power of attention to model the global context to obtain global semantic information. To capture the semantic dependencies between differences, we introduce a global attention module based on the self-attention mechanism^22^. Different from the self-attention mechanism, a global pooling of the feature map *R* is performed to obtain the global position encoding, after encoding the latter feature map *R* pays more attention to the relationship between related feature layers. Therefore, the output features of the global attention module will have clear semantics and contain context dependencies on surrounding objects.

**Figure 3:**
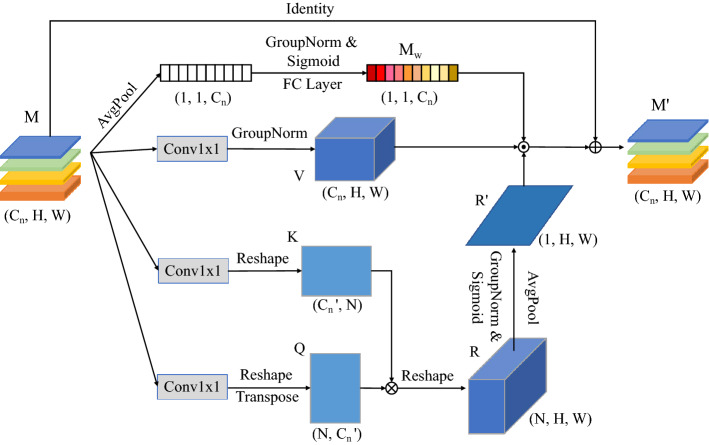
Architecture of semantic enhancement module.

As shown in Fig. 3, the shape of the global context *M* is $C_n \times H \times W$, we use the convolutional layer $W_q$ and $W_k$ respectively to transform it into a potential space. The transformed feature map is

4 $$\begin{aligned} Q= & {} W_qM, \end{aligned}$$

5 $$\begin{aligned} K= & {} W_kM, \end{aligned}$$

the shape of $\{Q, K\}$ is $C_n^{'} \times H \times W$. We reshape *Q* as $C_n^{'} \times N$, where $N=H \times W$. To obtain the relationship between different feature layers, we compute a correlation matrix as *R*, where *R* has the shape $N \times N$, and then reconstruct it as $N \times H \times W$. After normalizing the *R* group, going through sigmoid and average pooling, we build a matrix $R^{'}\in \mathbb {R}^{1 \times H \times W}$

6 $$\begin{aligned} R^{'}=\sigma (AvgPool(R))=\sigma (AvgPool(Q^TK), \end{aligned}$$

at the same time, we utilize the convolutional layer $W_v$ to transform the feature map *M* into another representation *V*:

7 $$\begin{aligned} V=W_vM, \end{aligned}$$

the shape of *V* is $C_n \times H \times W$. Finally, the feature maps *V* and $R^{'}$ are multiplied by the dot-product, then element-wise product is performed on $M_w$, and then the feature map *M* is added to it to obtain global semantic $M^{'}$, and we express the function as

8 $$\begin{aligned} M^{'}=(R^{'}\odot V *M_{w})+M, \end{aligned}$$

which $\odot$ represent dot-product, $*$ represent element-wise product.

In the semantic enhancement module, the information between each channel is exchanged and the channel information is weighted and modeled in a global context using channel-space attention to enhance the semantic information.

### Semantic injection module

After obtaining the global semantic information, we directly fuse it with the original feature maps $\{C_2, C_3, C_4, C_5\}$ to avoid the semantic information being thinned out in the top-down feature fusion process. However, a significant semantic gap exists between the original feature layer and the global semantic, and a simple 1 $\times$ 1 convolutional layer would not effectively fuse the global semantic information with the original feature layer. Therefore, we introduce a semantic injection module (SIM) to alleviate the semantic gap.

**Figure 4:**
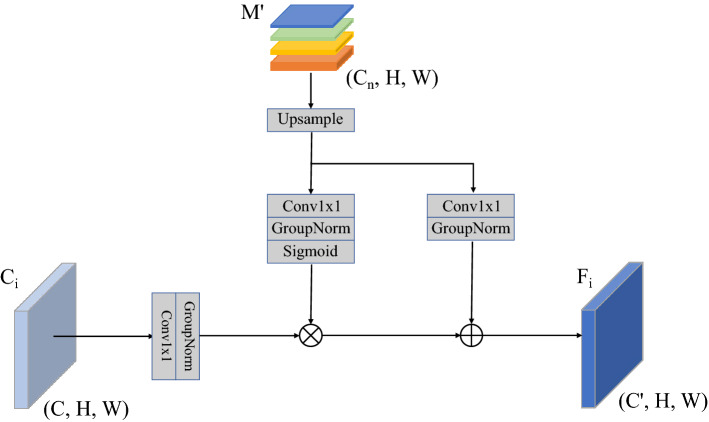
Illustration of semantic injection module.

As shonw in Fig. 4, the SIM takes the original feature maps $\{C_2, C_3, C_4, C_5\}$ and the global semantic as input. The original feature maps $\{C_2, C_3, C_4, C_5\}$ generate the features to be injected through the 1 $\times$ 1 convolutional layer, the global semantic is sent to the 1 $\times$ 1 convolutional layer, and then the group normalization layer and the sigmoid layer are input to generate the semantics at the same time, the global semantic also go through a 1 $\times$ 1 convolutional layer and then normalized. The three outputs have the same 256 channels and size. Then, the original feature maps $\{C_2, C_3, C_4, C_5\}$ are injected with global semantics in the form of matrix multiplication, and global semantics are added to new features after injection. After semantic injection, the original features at each level can obtain semantic information and localization information from the feature maps at different scales, which alleviates the semantic gap between different feature levels.

### Gated channel guidance module

There are semantic differences in the mapping of features across scales, and fusing features between scales can lead to confounding effects, confusing localization and recognition^7^. The proposed SIM incorporates more cross-scale feature mappings, resulting in more severe aliasing effects than the original FPN. To reduce the aliasing effect’s negative impacts, inspired by the gating unit^14^, we propose a Gated Channel Guidance Module (GCG) to selectively accept semantic information contained in the features from the higher-level features, further refining the fused features to make them more discriminative.

**Figure 5:**
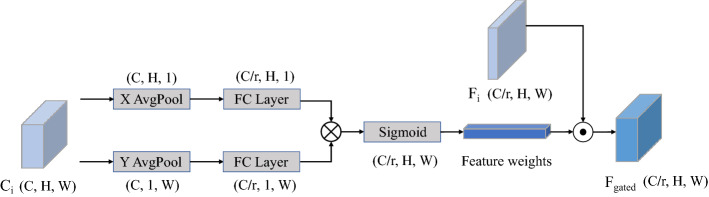
Illustration of gated channel guidance module (GCG).

The feature map $C_i(i=2,3,4,5)$ is partitioned into two directions of width and height as illustrated in Fig. 5, the pooling kernel size is (H, 1) and (1, W) to perform global average pooling on the feature maps to obtain $F_h$ and $F_w$,

9 $$\begin{aligned} F_{h}=AvgPool((H,1))\ and\ F_{w}=AvgPool((1,W)), \end{aligned}$$

next, $F_h$ and $F_w$ are passed to fully connected layers respectively, and the feature weights are obtained by the sigmoid function. Finally, the dot-product of $F_i$ and feature weights get $F_gated$. This process can be expressed as

10 $$\begin{aligned} F_{gated}=\sigma (fc_{1}(F_{h}) \otimes fc_{2}(F_{w}) \odot F_{i}, \end{aligned}$$

which $\sigma$ represents the sigmoid activation function, $\otimes$ represents matrix multiplication, $\odot$ represents dot-product operator, $fc_1$ and $fc_2$ represent a fully connected operation, and i denotes the pyramid levels index.

## Experiments

### Dataset and evaluation metrics

All our experiments are implemented on the MS COCO^25^ dataset, which contains 80 categories. It has 115 k images for training (train-2017) and 5 k images for validation (val-2017). There are also 20 k unlabeled images in test-dev. We describe ablation investigations and final results for val-2017 and test-dev after training the model on train-2017. All results were reported following typical COCO-style Average Precision (AP) measurements.

**Table 1:** Comparison with baseline and state-of-the-art methods developed for COCO test.

Method	Backbone	Schedule	AP	$AP_{50}$	$AP_{75}$	$AP_S$	$AP_M$	$AP_L$
Baseline								
RetinaNet$^*$	ResNet-50-FPN	1 $\times$	36.3	55.5	38.7	20.5	40.1	47.5
Faster R-CNN$^*$	ResNet-50-FPN	1 $\times$	37.3	58.3	40.3	21.2	40.8	48.2
Faster R-CNN$^*$	ResNet-101-FPN	1 $\times$	39.3	60.3	43.1	22.3	43.4	49.9
Faster R-CNN$^*$	ResNet-101-FPN	2 $\times$	39.9	60.3	43.2	23.0	43.8	52.9
Faster R-CNN$^*$	ResNext-101-32x4d-FPN	1 $\times$	40.5	62.8	44.0	24.3	43.9	50.2
Faster R-CNN$^*$	ResNext-101-64x4d-FPN	1 $\times$	41.8	64.4	45.6	24.7	46.1	54.2
State-of-the-art								
RetinaNet	ResNet-50-AugFPN	1 $\times$	37.5	58.4	40.1	21.3	40.5	47.3
Faster R-CNN	ResNet-50-AugFPN	1 $\times$	38.8	61.5	42.0	23.3	42.1	47.7
Faster R-CNN	ResNet-101-AugFPN	1 $\times$	40.6	63.2	44.0	24.0	44.1	51.0
Faster R-CNN	ResNet-101-AugFPN	2 $\times$	41.5	63.9	45.1	23.8	44.7	52.8
Faster R-CNN$^*$	ResNext-101-32x4d-AugFPN	1 $\times$	41.9	64.4	45.6	25.2	45.4	52.6
Faster R-CNN$^*$	ResNext-101-64x4d-AugFPN	1 $\times$	43.0	65.6	46.9	26.2	46.5	53.9
Libra RetinaNet$^*$	ResNet-50-FPN	1 $\times$	37.8	57.5	40.5	21.5	40.8	47.4
Libra R-CNN$^*$	ResNet-50-FPN	1 $\times$	38.6	60.0	42.0	22.4	41.3	47.7
Libra R-CNN$^*$	ResNet-101-FPN	1 $\times$	40.2	61.2	44.1	22.7	43.6	52.1
Libra R-CNN$^*$	ResNet-101-FPN	2 $\times$	41.0	62.0	44.7	23.3	43.9	52.6
Libra R-CNN$^*$	ResNext-101-32x4d-FPN	1 $\times$	41.8	62.9	45.7	24.3	44.8	52.8
Libra R-CNN$^*$	ResNext-101-64x4d-FPN	1 $\times$	43.0	64.2	46.9	25.2	45.9	54.1
Ours								
RetinaNet	ResNet-50-MSEFPN	1 $\times$	**37.7**	57.5	40.2	21.2	40.9	46.9
Faster R-CNN	ResNet-50-MSEFPN	1 $\times$	**39.4**	61.7	42.2	22.5	41.7	48.7
Faster R-CNN	ResNet-101-MSEFPN	1 $\times$	**41.2**	63.2	44.3	23.6	44.5	52.0
Faster R-CNN	ResNet-101-MSEFPN	2 $\times$	**41.6**	63.5	44.7	23.3	44.5	53.2
Faster R-CNN	ResNet-101-32x4d-MSEFPN	1 $\times$	**42.2**	64.2	45.6	24.3	45.4	53.2
Faster R-CNN	ResNet-101-64x4d-MSEFPN	1 $\times$	**43.4**	65.5	46.8	25.7	46.6	54.5

The highest AP in the same class of models is in [bold].

The symbol ‘$*$’ represents the result we re-implemented by mmdetection.

### Implementation details

For fair comparisons, all experiments are implemented based on mmdetection^26^. We resize the image input to (1333, 800). In the process of training, the $1\times$ schedule denotes 12 epochs and 24 epochs for the $2\times$ schedule. The models are trained on 8 GPUs (2 images per GPU). The initial learning rate defaults to 0.02 for the $1\times$ schedule, the learning rate drops by a factor of 0.1 after the 8th and 11th rounds, respectively, and for the $2\times$ schedule, it drops by a factor of 0.1 after the 16th and 22nd rounds. If not specifically stated, other hyper-parameters follow the mmdetection basic settings.

### Main results

In this section, the evaluation of MSE-FPN is operationalized on the COCO test development set and compared to other state-of-the-art detectors. Since mmdetection has been upgraded to version 2.0, we have re-implemented the corresponding baseline method for FPN in mmdetection for a fair comparison. As Table 1 demonstrates, For the single-stage detector RetinaNet, we observed a significant improvement in performance from 36.3 AP to 37.7 AP when using MSE-FPN. When Faster R-CNN using ResNet50 as the backbone was employed, the performance improved to 39.4 AP by replacing FPN with MSE-FPN, which is 2.1 points higher than Faster R-CNN based on ResNet50-FPN. We also evaluated our proposed method with ResNext101-32x4d and ResNext101-64x4d as feature extractors, and the FPN-based method achieved 40.5/41.8 AP, while MSE-FPN achieved 42.2/43.4 AP. These results demonstrate that MSE-FPN can improve the performance of even stronger backbone networks. Moreover, our model also brings an overall improvement for $AP_S$, $AP_M$, $AP_L$ (AP results for small, medium and large objects respectively), especially for $AP_S$, which demonstrates the effectiveness of our proposed method in capturing features useful for the detection task. All the improvements show that our MSE-FPN is effective.

Furthermore, we compared MSE-FPN with other state-of-the-art detectors. However, due to mmdetection v2.0 performs better than version 1.0, we re-implemented Libra R-CNN^8^ and AugFPN^9^ on mmdetection v2.0 for a fair comparison. When compared to the data in the Libra R-CNN study, the final performance of our re-implemented results is similar. As shown in Table 1, when using ResNet-101 as the backbone network and 1$\times$ Schedule, the MSE-FPN-based Faster R-CNN obtained 41.2 AP, while the Libra R-CNN and AugFPN obtained 40.2 AP and 40.6 AP, respectively. At 2$\times$ schued fully trained, MSE-FPN obtained 41.6 AP, Libra R-CNN and AugFPN obtained 41.0 AP and 41.5 AP respectively, Libra R-CNN has not yet reached the performance of MSE-FPN at 1$\times$ schued, while AugFPN has only 0.3 AP more than MSE-FPN at 1$\times$ schued, which validates that our method does not require a lot of training time to achieve more satisfactory results. In summary, our experimental results show that MSE-FPN can achieve competitive performance to the state-of-the-art detectors such as Libra R-CNN and AugFPN.

**Table 2:** The effect of each component in MSE-FPN is tested on COCO val-2017.

SEM	SIM	GCG	AP	$AP_{50}$	$AP_{75}$	$AP_S$	$AP_M$	$AP_L$
			36.9	57.8	39.7	20.5	40.7	47.5
$\surd$			38.1	58.9	40.8	21.6	41.2	48.2
	$\surd$		38.2	59.2	41.1	21.4	41.1	48.1
		$\surd$	38.1	59.1	41.3	21.5	41.3	48.3
$\surd$	$\surd$		38.8	60.1	41.9	21.9	41.4	48.4
$\surd$		$\surd$	38.6	59.9	41.7	21.7	41.2	48.1
	$\surd$	$\surd$	38.5	59.7	41.6	21.5	41.1	48.2
$\surd$	$\surd$	$\surd$	**39.4**	61.7	42.2	22.5	41.7	48.7

The highest AP is in [bold].

*SEM* semantic enhancement module, *SIM* semantic injection module, *GCG* gated channel guidance module.

### Ablation experiments

We conducted ablation experiments to evaluate the significance of each component of MSE-FPN, and the overall results of the ablation experiments are shown in Table 2. We gradually added the SEM, SIM, and GCG to the ResNet50-FPN FasterR-CNN baseline. Since SEM and SIM are cooperative, when one of the modules is used separately, the other is simply replaced with a $1\times 1$ convolutional layer, and the training process followed a $1\times$ schedule^26^ (12 epochs). Ablation experiments are performed with the same settings for a fair comparison.

**Table 3:** Ablation experiments accepting feature layers of different scales.

Input	AP	Params (M)	FLOPS (G)
{1/4 1/8 1/16 1/32}	39.6	38.24	252.84
{1/8 1/16 1/32}	39.3	37.67	250.24
{1/16 1/32}	38.7	37.52	249.61

#### Ablation studies on semantic enhancement module

In this section, we discuss the SEM from two aspects: accepting input from different scale feature layers M, and the residual attention module in the SEM. Table 3 shows the results of experiments conducted with feature layers from different scales as inputs to the SEM. We found that the best performance is achieved using feature maps from $\{1/4, 1/8, 1/16, 1/32\}$ with the most amount of computation. With the feature maps from $\{1/16, 1/32\}$, the computation is minimal but the performance is the worst. In all other experiments, we chose feature maps from $\{1/8, 1/16, 1/32\}$ to achieve a balance between accuracy and computational cost. As shown in Table 4, we tested the SEM using three schemes. For fairness, we changed the output feature map channel number by adding 1 $\times$ 1 convolutional layers after the SEM output. (a) shows that using the spatial attention module alone, AP improves by 0.7, and (b) shows that using the channel attention module alone, AP improves by 0.8. (c) represents a combination of scheme a and scheme b improves AP by 1.2. Scheme c achieves a more compelling performance compared to the baseline. The experimental demonstrated that our proposed SEM can fully utilize the channel and spatial information brought by the original feature layer to facilitate the object detection task.

**Table 4:** Ablation experiments of semantic enhancement module.

Methods	AP	$AP_{50}$	$AP_{75}$	$AP_S$	$AP_M$	$AP_L$
Baseline	36.9	57.8	39.7	20.5	40.7	47.5
Baseline + $SEM_a$	37.6	58.6	40.5	21.4	40.9	47.8
Baseline + $SEM_b$	37.8	58.5	40.4	21.3	40.8	47.7
Baseline + $SEM_c$	38.1	58.9	40.8	21.6	41.2	48.2

#### Ablation studies on semantic injection module

As shown in Table 5, the AP value of SIM alone is 1.3 higher than the baseline value (using 1 $\times$ 1 conv), and Table 2 shows that the AP is improved by 1.9 when SIM and SEM are used together. These results indicate that the two modules are closely related and complement each other.

**Table 5:** Ablation experiments with semantic injection module and $1\times 1$ conv.

SIM	$1\times 1$ conv	AP	$AP_{50}$	$AP_{75}$	$AP_S$	$AP_M$	$AP_L$	Params (M)	FLOPS (G)
$\surd$		38.2	59.2	41.1	21.4	41.1	48.1	36.74	248.34
	$\surd$	36.9	57.8	39.7	20.5	40.7	47.5	35.88	247.69
$\surd$	$\surd$	38.3	59.4	41.3	21.3	41.2	48.2	37.42	249.53

In this section, we discuss the effect of SIM with $1\times 1$ convolutional layer on MSE-FPN. The results presented in Table 5 demonstrate that using $1\times 1$ conv alone leads to a relatively low AP value, indicating that traditional $1\times 1$ convolutional layers may lose some channel information when reducing dimensions. When we add a $1\times 1$ convolutional layer before the SIM module, it has the same AP value as using the SIM module alone. Therefore, SIM+$1\times 1$ conv does not affect network performance, but it increases the computational cost and network parameters. This also shows that our method fully utilizes the channel information from the $C_i$ feature layer and the global semantic features obtained through SEM with only a small increase in parameters and computation, further narrowing the semantic gap between different feature layers, and effectively replacing the traditional $1\times 1$ convolutional layer.

#### Ablation studies on gated channel guidance module

To mitigate the effect of aliasing, GCG weighted the fusion of features at different levels before feature fusion, using gating units to selectively receive information from features at different scales to mitigate feature confusion. According to Table 6, the proposed GCG method achieves an improvement of 1.2 AP in performance compared to the baseline.

We also investigate the different effects of different attention configurations through ablation experiments. First, We replaced gated channel guidance module(GCG) with channel attention guided module(CAG)^10^ and coordinate attention(CA)^22^ respectively before feature fusion. As shown in Table 6, CAG is less computationally intensive as it only uses a simple linear layer to refine the channel features, but the performance gain is less pronounced at 0.7 AP due to the lack of focus on spatial information, while the CA embeds positional information into the channels and the performance gain is more significant with 0.9AP improvement, but the large number of convolution and pooling operations leads to a slight increase in computation. Our GCG only uses pooling operations and linear mapping to embed location information into channels, and then uses a simple gating unit to selectively retain some information useful for the detection task, which ensures performance (1.2 AP improvement compared to the baseline) and reduces computation. We can also see from the other metrics in Table 6 that GCG outperforms the other modules.

**Table 6:** Ablation experiments of different attention modules of COCO val-2017.

Setting	AP	$AP_{50}$	$AP_{75}$	$AP_S$	$AP_M$	$AP_L$	Params (M)	FLOPS (G)
Baseline	36.9	57.8	39.7	20.5	40.7	47.5	35.32	247.18
CAG^10^	37.6	58.7	40.8	20.7	40.8	47.9	37.18	248.55
CA^22^	37.8	58.9	41.0	21.1	41.1	48.0	38.56	249.68
GCG	38.1	59.1	41.3	21.5	41.3	48.3	37.21	249.87

## Conclusion

In this paper, we analyze the FPN intrinsic issues and discover that there is a large semantic gap between different feature layers, directly fusing these features will result in feature confusion, and the original multi-scale features are not fully utilized, more channel information will be lost in the dimensionality reduction of advanced features. To tackle these issues, we propose a novel multi-scale semantic enhanced feature pyramid network (MSE-FPN). It consists of three simple yet effective components, specifically, we use the semantic enhancement module (SEM) to extract global semantic information and feed it into the semantic injection module (SIM) to narrow the semantic gap between different feature layers and alleviate the loss of channel information, and then the gated channel guidance module (GCG) is introduced to alleviate the aliasing effect between different feature layers. Experiments show that MSE-FPN can substantially improve baseline methods on the challenging MS COCO dataset.

## Data Availability

The datasets used during the study are available at https://cocodataset.org.

## Abbreviations

FPN: Feature pyramid network
AugFPN: Augmented FPN
CEFPN: Channel enhancement FPN
BiFPN: Bi-directional FPN
PAFPN: Path aggregation FPN
ACFPN: Attention-guided context FPN
MSE-FPN: Multi-scale semantic enhancement FPN
CNN: Convolutional neural networks
R-CNN: Region-CNN
Fast R-CNN: Fast region-CNN
Faster R-CNN: Faster region-CNN
Cascaded R-CNN: Cascaded region-CNN
Libra R-CNN: Libra region-CNN
Mask R-CNN: Mask region-CNN
SPPNet: Spatial pyramid pooling networks
ResNet: Deep residual network
RPN: Region proposal network
YOLO: You only look once
SSD: Single shot multibox detector
SEM: Semantic enhancement module
SIM: Semantic injection module
GCG: Gated channel guidance module
FC: Fully connected operation
AvgPool: Average pooling
RSCTIR: Remote sensing text-image retrieval
MIDF: Multi-level information dynamic fusion
MCRN: Multi-source cross-modal retrieval network
GMU: Gated multimodal units
